# IgA and Neutralizing Antibodies to Influenza A Virus in Human Milk: A Randomized Trial of Antenatal Influenza Immunization

**DOI:** 10.1371/journal.pone.0070867

**Published:** 2013-08-14

**Authors:** Elizabeth P. Schlaudecker, Mark C. Steinhoff, Saad B. Omer, Monica M. McNeal, Eliza Roy, Shams E. Arifeen, Caitlin N. Dodd, Rubhana Raqib, Robert F. Breiman, K. Zaman

**Affiliations:** 1 Cincinnati Children's Hospital and Medical Center, Division of Infectious Diseases, Global Health Center, Cincinnati, Ohio, United States of America; 2 Rollins School of Public Health, Emory University, Atlanta, Georgia, United States of America; 3 International Centre for Diarrheal Disease Research, Bangladesh, Dhaka, Bangladesh; 4 United States Centers for Disease Control and Prevention, Nairobi, Kenya; National Institutes of Health, United States of America

## Abstract

**Background:**

Antenatal immunization of mothers with influenza vaccine increases serum antibodies and reduces the rates of influenza illness in mothers and their infants. We report the effect of antenatal immunization on the levels of specific anti-influenza IgA levels in human breast milk. (ClinicalTrials.gov identifier NCT00142389; http://clinicaltrials.gov/ct2/show/NCT00142389).

**Methods and Findings:**

The Mother's Gift study was a prospective, blinded, randomized controlled trial that assigned 340 pregnant Bangladeshi mothers to receive either trivalent inactivated influenza vaccine, or 23-valent pneumococcal polysaccharide vaccine during the third trimester. We evaluated breast milk at birth, 6 weeks, 6 months, and 12 months, and serum at 10 weeks and 12 months. Milk and serum specimens from 57 subjects were assayed for specific IgA antibody to influenza A/New Caledonia (H1N1) using an enzyme-linked immunosorbent assay (ELISA) and a virus neutralization assay, and for total IgA using ELISA. Influenza-specific IgA levels in breast milk were significantly higher in influenza vaccinees than in pneumococcal controls for at least 6 months postpartum (*p* = 0.04). Geometric mean concentrations ranged from 8.0 to 91.1 ELISA units/ml in vaccinees, versus 2.3 to 13.7 ELISA units/mL in controls. Virus neutralization titers in milk were 1.2 to 3 fold greater in vaccinees, and correlated with influenza-specific IgA levels (r = 0.86). Greater exclusivity of breastfeeding in the first 6 months of life significantly decreased the expected number of respiratory illness with fever episodes in infants of influenza-vaccinated mothers (*p* = 0.0042) but not in infants of pneumococcal-vaccinated mothers (*p* = 0.4154).

**Conclusions:**

The sustained high levels of actively produced anti-influenza IgA in breast milk and the decreased infant episodes of respiratory illness with fever suggest that breastfeeding may provide local mucosal protection for the infant for at least 6 months. Studies are needed to determine the cellular and immunologic mechanisms of breast milk-mediated protection after antepartum immunization.

**Trial Registration:**

ClinicalTrials.gov NCT00142389

## Introduction

Young infants (0–6 months of age) throughout the world experience high rates of influenza infection, clinic visits, and hospitalization [1]–[6]. In some winters, as many as nine percent of all infants less than 6 months of age experience an influenza-related illness and require care in a clinic, emergency room or hospital ward [2]. Hospitalization rates for infants 0 to 6 months of age in the United States range from 45 to 104 per 10,000 infants. Of U.S. children less than age five who are hospitalized for influenza, 48% are infants less than 6 months of age [2], [6].

Despite the demonstrated burden of influenza illness, influenza immunization is not licensed for infants less than 6 months of age by the U.S. Federal Drug Administration. Oseltamivir was approved for use in infants greater than 2 weeks of age in December 2012. Few studies have been done to assess the safety and immunogenicity of influenza immunization in infants before 6 months of age [7], [8]. These studies suggest that influenza immunization in this age group produces low seroresponses, in part due to presence of passive maternal antibody [7], [8]. With limited prevention and treatment strategies, alternate approaches, including maternal immunization, are needed to protect infants from influenza during the 0 to 6 month period of high vulnerability.

Maternal antenatal immunization produces substantial levels of maternal and infant serum IgG [9], but we are not aware of reports describing the effect of antenatal immunization on specific anti-influenza IgA levels in human breast milk during prolonged lactation. Non-randomized observational studies suggest that breastfeeding is associated with protection against all respiratory disease [10], though data are limited with regard to specific influenza protection. A recent study in Argentina demonstrated that breastfeeding is associated with type I interferon production in infants infected with influenza virus [11]. A report of unvaccinated women from Bulgaria in 1994 used hemagglutination inhibition (HAI) assays to show that the presence of influenza antibody levels are somewhat higher in breast milk than in serum [12]. There have been studies in guinea pig milk [13] demonstrating protection of the pups when the mothers were immunized antepartum, as well as studies in humans describing breast milk antibody production for other microorganisms [14], [15].

Several studies have demonstrated that influenza immunization of mothers protects both mothers and young infants [16]–[19]. A unique randomized controlled trial of antenatal maternal immunization in Dhaka, Bangladesh demonstrated a 63 percent reduction in laboratory-proven influenza illness among infants born to influenza vaccinated mothers [19]. This clinical effectiveness was observed in infants up to 6 months of age, despite a steady decline in infant passive influenza serum IgG antibody after delivery resulting in very low levels at 5 to 6 months [9]. A retrospective study from the Navajo and White Mountain Apache Indian reservations in Southwest United States demonstrated a 41 percent reduction in the risk of laboratory-proven influenza infection for infants born to influenza-vaccinated women [16]. This study similarly showed a steady decline of passively acquired maternal anti-influenza antibody levels in infant serum [16]. Recent retrospective reports of antenatal influenza immunization from the New Vaccine Surveillance Network and from the northeastern United States show vaccine-associated reductions of laboratory-confirmed, influenza-associated hospitalization in less than 6 month old infants ranging from 45 to 92 percent, respectively [17], [18].

The steady decline in passively acquired infant serum anti-influenza IgG antibodies is in contrast with evidence of clinical protection of infants against influenza up to 6 months post-delivery. We hypothesize that specific anti-influenza IgA antibodies in breast milk may be sustained for 6 to 12 months post-delivery, potentially providing local mucosal protection for the infant during this time period. Our aim was to assess breast milk anti-influenza IgA antibody and virus neutralizing activity in influenza vaccine recipients and control mothers during 12 months of observation, and we demonstrated sustained levels of specific anti-influenza IgA in milk and decreased infant respiratory illness with fever for at least 6 months postpartum.

## Methods

### Ethics statement

The project protocol was reviewed and approved by the Ethical Review Committee at the International Centre for Diarrheal Disease Research, Bangladesh, the Institutional Review Board at the Bloomberg School of Public Health at Johns Hopkins University, Baltimore, and the Institutional Review Board at Cincinnati Children's Hospital Medical Center in Cincinnati, Ohio. Use of study vaccines was approved by the Directorate of Drug Administration, the Government of the People's Republic of Bangladesh.

### Study design and participants

**Figure 1:**
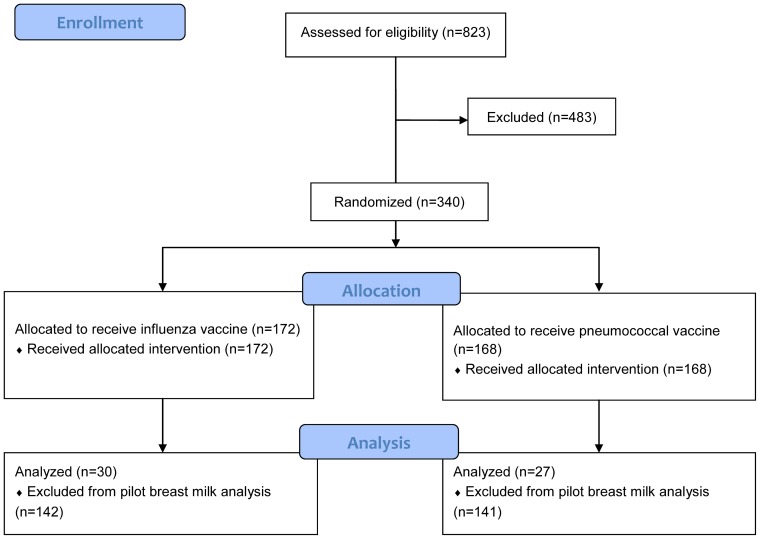
CONSORT study diagram. Of the 340 women randomized to receive influenza vaccine or pneumococcal vaccine, breast milk samples from 57 women were analyzed for IgA antibody and neutralizing activity.

The protocol for this trial and supporting CONSORT checklist are available as supporting information; see Checklist S1 and Protocol S1. The design, clinical methods and statistical analyses have been described for a prospective, individually randomized, double-blinded, parallel group trial (ClinicalTrials.gov number, NCT00142389, http://clinicaltrials.gov/show/NCT00142389), called the Mother'sGift Study. This study assessed the safety and immunogenicity of pneumococcal vaccines, as well as the clinical effectiveness of influenza vaccine, in Bangladeshi women and their infants followed for one year from delivery after immunization during the third trimester with either inactivated trivalent influenza vaccine or 23-valent pneumococcal polysaccharide (23vPPS) vaccine (control) [19]. We conducted a pre-specified secondary analysis to assess the concentration and duration of breast milk anti-influenza IgA antibody in mothers who received influenza vaccine during third trimester compared to control group mothers (Figure 1).

Briefly, mothers were recruited for the Mother'sGift study [19] at three clinics in Dhaka, Bangladesh, during the third trimester of pregnancy. After obtaining written informed consent, we randomly assigned 340 pregnant women aged 18–36 to receive either influenza or 23vPPS vaccine during the third trimester of pregnancy. Mothers reported breastfeeding frequency, along with frequency of other infant foods and episodes of infant respiratory illness with fever, every week for the first 6 months of life.

### Randomization and masking

The randomization sequence was computer-generated, stratified according to clinic, and blocked in groups of four; sequentially numbered opaque envelopes with data regarding assignments to study groups were provided to each clinic. Mothers, families, and study staff who collected data were unaware of the study-group assignments. Clinic staff members who were not involved with study-outcome assessments administered all doses of vaccine.

### Study Vaccines

Mothers were randomly assigned to receive the inactivated influenza virus vaccine, Fluarix®, containing the WHO recommended influenza antigens for the southern hemisphere in 2004: A/New Caledonia (H1N1), A/Fujian (H3N2), and B/Hong Kong (lot number, AFLUA004BC; GlaxoSmithKline Biologicals), or the 23vPPS vaccine, Pneumovax® (lot number, 0987N; Merck & Co., Inc.). All study vaccines were purchased from the manufacturer. The Fluarix® was given intramuscularly with a 1.5 centimeter needle, while the Pneumovax® was given subcutaneously with a 0.5 centimeter insulin syringe.

### Antibody Assays

Breast milk samples were collected by hand expression by all participants at delivery, and at 6, 10, 14, 18 weeks, and 6 and 12 months post-delivery. Maternal serum was obtained at 10 weeks and 12 months post-partum. For this analysis, we evaluated breast milk at birth, 6 weeks, 6 months, and 12 months, and maternal serum at 10 weeks and 12 months. The milk whey was separated and frozen at minus 70° Celsius. Specimens were transported to Cincinnati Children's Hospital Medical Center, where breast milk and serum from 57 subjects were evaluated for IgA specific for influenza A/New Caledonia (H1N1) antigen using an enzyme-linked immunosorbent assay (ELISA). Briefly, the ELISA was performed by coating plates with 0.75 µg/ml of baculovirus-expressed recombinant hemagglutinin derived from the A/New Caledonia/20/99 (H1N1) strain (BEVS rHA, Protein Sciences Corporation, Meriden CT). After an overnight incubation, ELISA plates were washed with PBS +0.05% Tween 20 and blocked with 0.89% BSA solution (Bovine Serum Albumin Fraction V 7.5% solution, Gibco-Invitrogen, Carlsbad, CA) in 1% non-fat dry milk (Carnation Nonfat Dry Milk, Nestle Food Company, Vevey, Switzerland). A human serum reference standard serially diluted to generate a reference curve and samples diluted at 1:20 and 1:200 were added to duplicate wells on each plate. The plates were incubated, washed and biotinylated rabbit-anti human IgA (Jackson ImmunoResearch Laboratories, West Grove, PA) was added. After incubation, the plates were washed and peroxidase conjugated avidin-biotin solution (Vector PK4000 Kit, Vector Laboratories, Burlingame, CA) was added. After incubation and final washing the substrate, O-phenylenediamine, dihydrochloride solution (OPD 15 mg tablet, Sigma Aldrich, St. Louis, MO) was added and incubated. The colorimetric reaction was stopped by adding 1 M sulfuric acid and the plates were read at a wavelength of 490 nm. The concentration of influenza-specific IgA was derived by extrapolation from the standard curve generated from the reference serum with the assigned quantity of anti-influenza IgA expressed in ELISA units/mL (EU). Samples below the limit of detection (0.04 EU) were assigned a value of <0.04. Samples that were above the limit of the standard curve were repeated at a higher dilution to obtain a reportable value.

We also performed a total IgA EIA assay that was similar to the above assay except that plates were coated with Rabbit anti-human IgA, and a purified human IgA (Sigma Aldrich, St. Louis, MO) was used as a standard. A serum neutralization assay for influenza A/New Caledonia (H1N1) on the 57 milk samples was performed, using a method previously described by Rowe et al [20] with some modifications. Using an extrapolation method created in SoftMax Version 5.3, the OD values of the dilution series of each sample was modeled using a five parameter logistic regression function. For each fitted curve, the dilution which corresponds to a 50% response is extrapolated. This value represents the titer of the serum against a given virus, which represents a 50% reduction in amount of virus.

### Statistical Analysis

The numbers of subjects that were needed for the primary study were calculated to detect a specified difference in mean pneumococcal antibody titer in the two groups [19]. A comparison of characteristics of mothers in this report was done. Continuous variables were summarized as mean and standard deviation and differences between groups were tested with a two-sample T-test. Categorical variables were compared across groups using a chi-squared test of independence. We calculated geometric mean concentrations (GMC) of anti-influenza IgA antibodies in breast milk, and of neutralizing titers for subjects at four time points. Differences between vaccine groups in log-transformed IgA titers were tested using a repeated measures analysis of variance. Due to the correlation among outcome measures in each family of comparisons, we used the Bonferroni method to adjust for multiple comparisons. Since breast milk composition, including total IgA, is known to vary by time of day, by time since last feeding, and during the feeding, we adjusted the specific IgA levels in each milk specimen by dividing specific IgA levels by total IgA to obtain an adjusted specific IgA level. This increased the likelihood that changes in influenza-specific IgA were secondary to type of vaccine received rather than routine variations in breast milk composition. In the breast milk analysis, the response variables for the repeated measures ANOVA models were antigen-specific IgA in breast milk, total IgA in breast milk, neutralization titers in breast milk, and adjusted specific IgA, while independent variables were maternal vaccine group and postpartum time. In the serum analysis, the response variables for the repeated measures ANOVA models were antigen-specific IgA and neutralization titers in serum, while independent variables were maternal vaccine group and time. We also calculated ratios of neutralization to IgA titers in milk and serum in vaccine and controls. In order to test the effect of breast milk on infants born to the 57 influenza- and pneumococcal- vaccinated mothers, we analyzed the effect of maternal vaccine and exclusivity of breastfeeding on infant episodes of respiratory illness with fever. We calculated a breastfeeding score as an average measure of exclusive breastfeeding for the first 6 months of life. Weeks of exclusive breastfeeding were given a score of 1, partial breastfeeding 0.5, and no breastfeeding 0. We then averaged the score over all weeks. We conducted a stratified Poisson regression analysis modeling the number of respiratory illness episodes in the first 6 months of life in each of the two vaccine groups.

## Results

Of 340 women enrolled in the study, 172 women received influenza vaccine and 168 received 23-valent pneumococcal capsular polysaccharide vaccine, of which 30 and 27, respectively, had both breast milk and serum available over 12 months for analysis. Differences between vaccine groups were non-significant for maternal age, birth length, birth weight, gestational weeks, maternal education, maternal height, Apgar scores, gravidity, parity, breastfeeding score, delivery type, and infant sex (Table 1). Differences were statistically significant for interval from vaccination to delivery, with mothers who received pneumococcal vaccine experiencing a 13 day longer interval than mothers who received influenza vaccine. Additionally, place of delivery was significantly different between groups, with mothers who received influenza vaccine delivering more frequently at a hospital (Table 1).

**Table 1:** Maternal and infant characteristics by vaccine group.

	VACCINE		
Variable	Influenza (n = 30)	Control (n = 27)	*p* value*
Maternal Age (years)	24.27±4.20	24.70±3.71	0.68
Birth length (centimeters)	49.52±1.81	49.15±2.02	0.46
Birth weight (kilograms)	3.04±0.38	3.14±0.52	0.39
Gestational weeks	39.17±1.32	39.48±1.34	0.38
Maternal Education (years)	11.33±3.23	11.44±2.82	0.89
Mother's height (centimeters)	152.88±4.68	153.33±5.69	0.75
Apgar at 1 minute	8.07±0.83	8.26±0.53	0.31
Apgar at 5 minutes	9.33±0.66	9.37±0.49	0.81
Gravidity	1.80±1.10	2.04±1.13	0.42
Parity	0.93±0.83^+^	1.13±0.72^++^	0.49
Breast feeding score (1 = 6 months of exclusive breast milk)	0.81±0.16	0.85±0.14	0.35
Days from vaccination to delivery	51.27±13.11	64.89±15.11	0.0006
**Place of Delivery**			0.02
Clinic	19 (63.33%)	24 (88.89%)	
Hospital	11 (36.67%)	3 (11.11%)	
**Delivery Type**			0.52
Normal	11 (36.67%)	8 (29.63%)	
Episiotomy	9 (30.00%)	6 (22.22%)	
Caesarean	10 (33.33%)	13 (48.15%)	
**Infant Sex**			0.44
Male	17 (56.67%)	18 (66.67%)	
Female	13 (43.33%)	9 (33.33%)	

* p values were calculated using a T test for all variables except Place of Delivery, Delivery Type, and Infant Sex (Chi Squared). ^+^ N = 14^++^ N = 16.

### Influenza-specific IgA and neutralization antibody in breast milk

**Figure 2:**
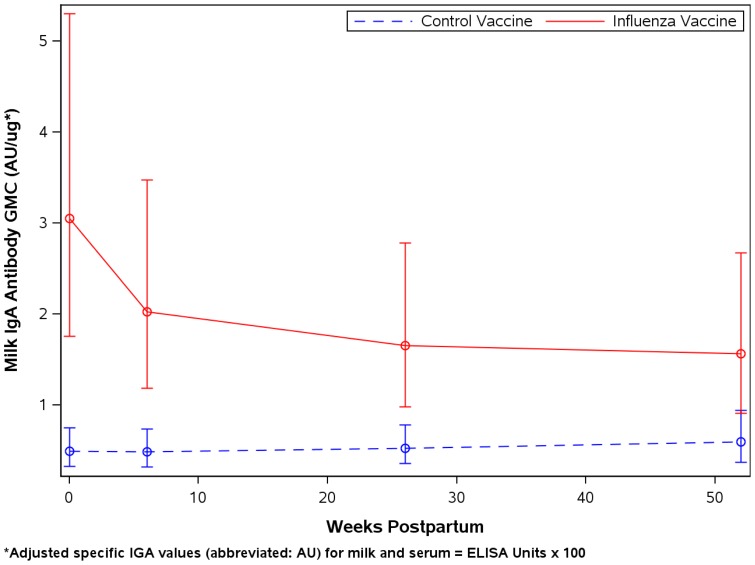
Geometric mean adjusted influenza-specific IgA in breast milk in influenza versus control vaccines. Geometric mean adjusted influenza-specific IgA antibody is significantly higher in the breast milk of influenza vaccinees compared to control vaccinees for at least 6 months postpartum.

Mean anti-influenza IgA antibody was higher in milk of the mothers who received influenza vaccine, with the highest levels at delivery (Table 2). After adjustment for multiple comparisons, anti-influenza IgA was significantly higher in influenza vaccinees at delivery, 6 weeks, and 6 months, but similar between groups at 12 months. In contrast, total IgA in breast milk was similar between vaccine groups at all four time points and dropped steadily over the study period. Neutralization titers were significantly higher in influenza vaccinees at the time of delivery but were not significantly different between groups at 6 weeks, 6 months, and 12 months postpartum. Adjusted specific IgA levels (Figure 2) were significantly greater in the influenza vaccine group and remained significantly greater for 6 months. The GMCs for specific anti-influenza IgA antibody in milk over all time points were 16.9 and 4.19 EU/ml in vaccinees and controls, respectively (*p*<0.0001). There was no significant statistical interaction between vaccine and time, meaning that the rate of decrease in serum or milk antibody was similar between study groups.

**Table 2:** Breast milk IgA antibodies and influenza virus neutralization titers in influenza and control vaccines.

		Influenza vaccine		Control vaccine				
Time postpartum	Assay	N	GMC (95% CI)	N	GMC (95% CI)	*p* value	Bonferroni Adjusted *p* value	Influenza/Control vaccine ratio
0 days	Influenza Specific IgA (EU/mL)	29	91.1 (48.6–170.8)	27	13.7 (7.8–24.3)	<0.0001	<0.0001	6.64
	Total IgA (EU/mL)	29	2989.8 (2189.8–4082.2)	27	2778.0 (1884.1–4095.9)	0.66	1	1.08
	Adjusted Specific IgA	29	3.1 (1.8–5.3)	27	0.5 (0.3–0.7)	<0.0001	<.0001	6.17
	Neutralization Titer	28	186.5 (121.2–286.9)	27	63.7 (43.6–93.0)	<0.0001	0.001	2.93
6 weeks	Influenza Specific IgA (EU/mL)	30	9.6 (5.6–16.6)	27	2.3 (1.5–3.4)	0.0002	0.005	4.26
	Total IgA (EU/mL)	30	474.7 (416.7–540.9)	27	464.3 (401.9–536.4)	0.89	1	1.02
	Adjusted Specific IgA	30	2.0(1.2–3.5)	27	0. 5 (0.3–0.7)	<0.0001	0.001	4.16
	Neutralization Titer	30	19.4 (14.3–26.2)	26	12.1 (10.4–14.2)	0.07	1	1.60
6 months	Influenza Specific IgA (EU/mL)	30	8.0 (4.2–15.0)	27	2.3 (1.6–3.5)	0.001	0.04	3.43
	Total IgA (EU/mL)	30	483.0 (386.4–603.7)	26	472.1 (410.6–542.7)	0.89	1	1.02
	Adjusted Specific IgA	30	1.7(1.0–2.8)	26	0. 5 (0.4–0.8)	0.001	0.03	3.12
	Neutralization Titer	29	18.6 (12.3–28.1)	27	15.9 (10.5–24.2)	0.55	1	1.17
12 months	Influenza Specific IgA (EU/mL)	30	11.1 (6.0–20.7)	27	4.3 (2.6–7.0)	0.01	0.34	2.61
	Total IgA (EU/mL)	30	711.7 (557.0–909.3)	27	716.2 (561.4–913.7)	0.97	1	0.99
	Adjusted Specific IgA	30	1.6 (0.9–2.7)	27	0. 6(0.4–0.9)	0.005	0.13	2.63
	Neutralization Titer	28	19.7 (12.9–30.1)	27	15.5 (10.7–22.3)	0.35	1	1.27

Abbreviations: N, number; CI, confidence interval; GMC, geometric mean concentration.

### Influenza-specific IgA and neutralization antibody in serum

**Figure 3:**
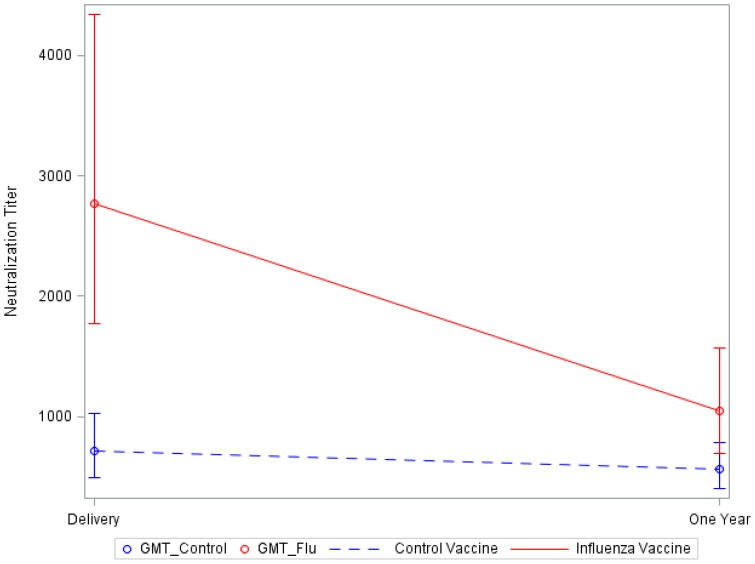
Geometric mean neutralization titers in serum in influenza versus control vaccines. Geometric mean anti-influenza neutralization titers are significantly higher in the serum of influenza vaccinees compared to control vaccinees at delivery.

Influenza-specific serum IgA levels and neutralization antibodies were higher in the mothers who received influenza vaccine, with the highest levels at delivery (Table 3). The influenza-specific IgA levels were statistically similar by 12 months postpartum. Neutralization antibodies were statistically different between groups at delivery (Figure 3), but titers were similar at 12 months postpartum after adjustment for multiple comparisons (alpha = 0.16). There was no significant interaction between vaccine and post-natal time, meaning that the rate of decrease in anti-influenza IgA antibody was similar between study groups.

**Table 3:** GMCs of serum influenza specific IgA antibody and influenza virus neutralization titers in influenza and control vaccines.

		Influenza vaccine		Control vaccine				
Time postpartum	Assay	N	GMC (95% CI)	N	GMC (95% CI)	*p* value	Bonferroni Adjusted *p* value	Influenza/Control vaccine ratio
0 days	Influenza Specific IgA (EU/mL)	30	29.2 (14.4–59.2)	26	9.2 (5.8–14.5)	0.01	0.04	3.12
	Neutralization Titer	30	2773.6 (1772.6–4339.9)	26	711.5 (493.7–1025.3)	<0.0001	<0.0001	3.88
12 months	Influenza Specific IgA (EU/mL)	28	235 (11.2–49.4)	26	10.3 (6.8–15.8)	0.06	0.35	2.25
	Neutralization titer	28	1046.4 (695.4–1574.6)	26	561.0 (401.0–784.8)	0.03	0.16	1.81

Abbreviations: N, number; CI, confidence interval; GMC, geometric mean concentration.

### Breastfeeding and infant episodes of respiratory illness with fever

Greater exclusivity of breast feeding in the first 6 months of life significantly decreased the estimated number of respiratory illness with fever episodes in infants of influenza-vaccinated mothers (*p* = 0.0042) but not in infants of pneumococcal-vaccinated mothers (*p* = 0.4154).

## Discussion

Our study examined breast milk and serum anti-influenza IgA antibody concentrations during the postpartum year after immunization of pregnant Bangladeshi women with either influenza or a control vaccine. To our knowledge, this is the first report of the levels and changes in human milk of specific anti-influenza IgA and of virus neutralization activity after antenatal influenza immunization. Mean breast milk antibody levels were significantly higher at delivery in mothers who received influenza vaccine and declined by six weeks, as has been described for other antigens [14], [15], [21], [22]. However, milk IgA concentrations specific for the seasonal A/New Caledonia (H1N1) antigen were statistically significantly higher in influenza vaccinees as long as 6 months postpartum. The higher levels of specific IgA and of adjusted specific IgA in influenza vaccinees through 6 months suggest active specific antibody production throughout lactation. This is further supported by the significantly decreased number of respiratory illness with fever episodes observed in infants of influenza-vaccinated mothers. We noted that the breast milk anti-influenza IgA GMCs in the vaccine and the control group increased between 6 months and 12 months postpartum, suggesting maternal natural antigenic stimulation from seasonal influenza in both groups. Total milk IgA concentrations were similar between vaccine groups and decreased by six weeks of age. However, adjusted anti-influenza specific milk IgA was statistically different for at least 6 months. Interestingly, postpartum time was not a significant predictor of adjusted specific anti-influenza milk IgA, meaning that the influenza-specific IgA was 2.6 to 6.6 times higher in influenza-immunized mothers for 6 months.

Our study had several potential limitations. The sample size was small, limiting our power to detect differences. We were unable to include a control group of women who were not breastfeeding, due to the difficulty of obtaining breast milk beyond the immediate postpartum period. Data from this South Asian setting may have limited generalizability to other regions with variations in influenza seasonality and exposure, and in maternal and infant nutritional status. We assessed immune responses to only a single antigen of the trivalent influenza vaccine, but patterns of sustained IgA milk antibody production are likely to be seen with other vaccine antigens [21], [22]. Previous analyses of serum HAI titers suggest a difference in antibody response and placental transfer of antibody by antigen [9].

The sustained high levels of specific IgA in breast milk of women immunized during pregnancy suggest that breastfeeding may provide vaccine-specific local mucosal protection for the infant up to 6 months of age [23], [24]. Secretory IgA is the most abundant immunoglobulin in breast milk, with concentrations of 1–2 g/L early in lactation [25]. It is secreted as a dimer linked via a secretory chain, conferring resistance against intestinal proteolysis [25]. We know from previous studies in women with vaccines against pertussis [26], rotavirus [27], and measles [28] that secretory IgA is produced in the breast milk in response to these vaccines. We are not aware of previous research documenting the production of specific anti-influenza IgA in breast milk after immunization. We are undertaking studies to determine the cellular and immunologic mechanisms of breast milk-mediated protection after antepartum immunization. These findings need replication, including with other vaccines like diphtheria, tetanus, and pertussis vaccine, but suggest that pregnant mothers should be aware of the infant benefits of influenza immunization pre- and post-partum. Few U.S. mothers breastfeed as long as 6 months [29], but the possibility of long-term protection of the infant from influenza should be considered in discussions with breastfeeding mothers.

## Supporting Information

Checklist S1 **CONSORT Checklist.**
(DOCX)

Protocol S1 **Trial protocol.**
(PDF)
