# Supplementary material for: IgA and Neutralizing Antibodies to Influenza A Virus in Human Milk: A Randomized Trial of Antenatal Influenza Immunization
Source: PLoS One. 2013 Aug 14;8(8):e70867. doi: 10.1371/journal.pone.0070867 (PMC3743877; doi:10.1371/journal.pone.0070867)

Revised Protocol

IMMUNOGENICITY OF CONJUGATED PNEUMOCOCCAL VACCINE IN INFANTS OF MOTHERS WHO HAVE OR HAVE NOT RECEIVED PNEUMOCOCCAL POLYSACCHARIDE VACCINE

Investigators:

Dr. Nigar S. Shahid, Professor M. C. Steinhoff

A Collaboration of:

Public Health Science Division,
ICDDR,B,
Dhaka, Bangladesh

Department of International Health,
School of Public Health,
Johns Hopkins University,
Baltimore, Maryland, USA

Submitted to:

Thrasher Fund
(Award # 02811-6 to Johns Hopkins University)

11 April 2002

Table of Contents

Page Numbers

Face Page.. 1

Project Summary... 3

Description of the Research Project ....

Hypothesis to be tested.. 5

Specific Aims 5

Background of the Project Including Preliminary Observations........................ 6

Research Design and Methods.. 8

Data Analysis... 14

Ethical Assurance for Protection of Human Rights...................................... 16

Collaborative Arrangements... 17

Budget Justifications.. 17

Dissemination and Use of Findings..17

Literature Cited.. 18

Appendix 1 Schedule of procedures... 20

Appendix 2 Summary of analyses 20

Appendix 3 Sample size tables... 21

Project Summary

In Bangladesh as in other developing countries, it is estimated that a quarter of the acute respiratory infections (ALRI) specific morbidity in children 0-4 years of age occurs in the first six months of life (1) and that 40-50% of the resulting ALRI mortality is due to pneumococcal infections (2). Fifty percent of these deaths occur in the neonatal period. Case management for ALRI is difficult because access to appropriate care is limited and antibiotic resistance is increasing (3). Current vaccine strategies and schedules fail to protect infections in the first months of life .

The rationale for this project is that in developing countries a substantial proportion of invasive pneumococcal disease occurs in infants before the age of 5 months. Currently available pneumococcal vaccines consist of purified capsular polysaccharides, and are effective in adults, but not in infants under 2 years of age. Glycoprotein conjugate pneumococcal vaccines are being developed which are immunogenic in infants. However, it is unlikely that they will be effective in preventing invasive disease before 4 or 5 months of age, after 2 or 3 doses have been administered starting at 6 weeks of age. The strategy of passive and active immunization of the infant has been successful in the reduction of neonatal and infant tetanus deaths, and UNICEF estimates that 40% of all pregnant women in developing regions currently receive tetanus toxoid. A similar strategy with pneumococcal vaccines may lead to reduction of invasive pneumococcal infections in infancy. However, because it is known that maternal antibodies inhibit the infant antibody response to measles, pertussis and tetanus vaccines, it is necessary to evaluate the biological feasibility of passive/active immunization against pneumococcal disease.

Earlier, we have immunized 75 mothers in Dhaka, Bangladesh and shown that maternal immunization results in relatively high titers of specific pneumococcal antibody in the newborn infant. We now propose to determine if maternal immunization will affect active immunization of the infant with conjugate pneumococcal vaccine.

We will evaluate the effect of pneumococcal immunization of pregnant women on the antibody response of the infants to glycoprotein conjugate pneumococcal vaccine. We will also examine the immune responses when the 3 dose regime of the glycoprotein conjugate pneumococcal vaccine is completed but without the maternal dose. Specifically, we will assess the effect of maternally acquired polysaccharide-specific antibody on the filial antibody response to glycoprotein conjugate pneumococcal vaccine, and the effect of maternally acquired carrier protein (diphtheria)-specific antibodies on the filial antibody response to conjugate pneumococcal vaccine and to active immunization with diphtheria vaccine.

Our methodology is a prospective randomized controlled trial to evaluate the effect of maternal immunization with pneumococcal vaccine on the filial antibody response to pneumococcal conjugate vaccine given at 6, 10 and 14 weeks of age. The primary outcome is the antibody titer to selected serotypes of pneumococcal vaccine and to tetanus toxoid. We will specifically compare the association between the levels of antibodies at birth and 6 weeks of age (passive maternal antibodies) with the level at 18-20 weeks of age (filial active antibody response) and at 9 months of age of the infant. As a secondary objective, we will determine the ability of infants in Dhaka to respond to new pneumococcal conjugate vaccine when given in the W.H.O. schedule without the

maternal dose. We will also assess the effect of the infants' serum IgG antibody and maternal IgA antibody in breast milk on colonization of the infants with pneumococci.

If we can show that the strategy of maternal + infant pneumococcal immunization to have a better immune response, then this strategy could be considered an appropriate immunization strategy for the regions to prevent early infant pneumococcal disease. Pneumococcal vaccines are licensed and readily available, and delivery systems to immunize pregnant women and infants are functioning in many developing countries. This strategy is operationally feasible and could have a substantial effect on early infant mortality.

KEY PERSONNEL (List names of all investigators including PI and their respective specialties)

Name	Professional Discipline/ Specialty	Role in the Project
ICDDR,B		
1. Dr Nigar S. Shahid	Epidemiology	Principal - Investigator
2. Dr Shams El Arifeen	Epidemiology	Co-investigator
3. Dr Rob Breiman	Epidemiology	Co-investigator
3. Dr Mahbubur Rahman	Microbiology	Co-investigator
4.To be identified	*Immunology*	*Co-investigator*
Johns Hopkins		
1. Dr Mark Steinhoff	Epidemiology	Co- principal - Investigator
2. Dr R . Hamilton	Immunology	Co-investigator
3.Dr A. Shankar	*Immunology*	*Co-investigator*

DESCRIPTION OF THE RESEARCH PROJECT

Hypothesis to be tested:

To determine the immunogenicity and safety of conjugate pneumococcal vaccine in infants of mothers who have been immunized with pneumococcal polysaccharide vaccine.

Specific Aims:

The specific objectives of the revised project are:

Primary Objectives

1. To assess whether the presence of maternally derived antibody to pneumococcal polysaccharide (or to the carrier protein) influences the immunogenicity and safety of glycoprotein conjugate pneumococcal vaccine when given to infants during routine immunization.

Secondary Objectives

1. To assess the effect of maternally-acquired polysaccharide-specific antibodies on the filial antibody response to glycoprotein conjugate pneumococcal vaccine.
2. To assess the effect of maternally-acquired carrier protein-specific antibodies on the filial antibody response to glycoprotein conjugate pneumococcal vaccine, and to active immunization with the carrier protein.
3. To determine the IgG antibody responses to conjugate pneumococcal vaccine when given at the standard EPI schedule to Bangladesh infants.
4. To assess the effect of specific serum IgG antibody acquired through maternal or infant immunization and of breast milk antibody on acquisition and nasopharyngeal carriage of pneumococci.

Background of the Project including Preliminary Observations

For developing countries diarrhoea and respiratory infections are two of the principle issues in child health (1,2). With the advent and the acceptability of the oral rehydration solution at the home level, the leading cause of childhood deaths is acute respiratory infections (ARI), accounting for nearly a fifth of all deaths below 5 years of age. The WHO algorithm for the treatment and case management of ARI is difficult because access to appropriate care is limited and antibiotic resistance is growing (3). Bacteria have an important role in severe and fatal pneumonia, especially in the developing world (4), where a half of children hospitalised with untreated severe pneumonia harbour bacteria in their lungs and *S. pneumoniae* and *H. influenzae* comprise up to 80% of the organisms isolated from the lungs of these patients (5). However, these two organisms can also be isolated from the upper respiratory tract as commensals in many infants without disease (6). However, these commensal organisms become pathogenic in the event of malnutrition and multiple infections. *S. pneumoniae*, also accounts for one of the two bacterial causes of severe invasive disease in the very young infant and causes up to 10% global annual deaths in infants < than 90 days of age (6). Results of the 1980's research programme supported by the Board on Science and Technology for International Development (BOSTID) and the recent WHO multi-center studies in Asia and Africa show that *S.pneumoniae* and *H.influenzae* are the major bacterial pathogens and that the respiratory syncytial virus (RSV) is the most common virus causing ALRI (4-8). Both the incidence of ARI and ALRI were high in children <18 months of age, with the highest being in early infancy (9). *S.pneumoniae* was the major pathogen in all groups studied, particularly in the first week of life (7). It was also the most important cause of meningitis; in children older than one week of age it accounted for 50% of all cases(6). Hence, any strategy aimed at preventing ARI deaths must include approaches to cover the period of most risk in the early infant. This means that interventions with ARI vaccines will probably have a greater impact the earlier in life they are given (11).Vaccination strategies to cover the period at most risk of infancy need to be evaluated (11).

The USFDA licensed 23-valent pneumococcal polysaccharide vaccine covers about 90% of circulating serotypes in Bangladesh (9). It produces high levels of antibody responses in adults (12). A recent study in Bangladesh showed that maternal immunization with the 23-valent polysaccharide pneumococcal vaccine, was safe and immunogenic and protective levels of antibodies (>0.15µg/mL) were observed in the infants' blood up to 22 weeks of age (13). Maternally transferred antibodies play an important role in infant protection, but the antibodies are short lived and may not fully cover the period of high risk. Maternal immunization does not produce impaired responses either with maternal anti-tetanus toxoid (TT) (a protein antigen) or with other polysaccharides as *H. influenzae* B (Hib) in infants (14). This may also induce the effect of priming in the newborn.

Vaccine efficacy depends on the prevalence of the serotypes in the vaccine, the age at which the children become immuno-competent to the predominant serotype, and the naturally acquired immunological response of the children to the important serotypes at the time of immunization (15). In developing countries, the serotypes that cause invasive disease are more likely to be ones that evoke a good immune to immunization in young children (16). Polysaccharide vaccines co-valently linked to a carrier protein are immunogenic in young infants from an early age(15). Clinical trials of the 2,5,7 & 9- valent glycoprotein conjugate pneumococcal vaccine have been conducted in young children both in developing and in developed countries and found to be safe and immunogenic when given along with routine immunization

(17- 23). The serotypes in the 7 –valent CRM $_{197}$ product would cover ~`60% of circulating serotypes here (24).

The proposal is to conduct a prospective randomized, blinded, controlled comparative trial of safety and immunogenicity of 2 US FDA licensed pneumococcal vaccines. Women will be immunized around 32 weeks gestation and infants at birth. To-date no studies have examined the immunogenicity and safety of the conjugate pneumococcal vaccine with maternal immunization.. Globally early infant deaths remain a significant problem, which cannot be addressed with current EPI strategies, even with new vaccines. This project builds on results of a previous study and will build capacity for vaccine evaluation in Bangladesh.

RESEARCH DESIGN AND METHODS:

Study Design:

A randomized double-blind controlled immunogenicity trial will be conducted in Dhaka in collaboration with private and public facilities providing maternity health care services. In this study mothers will be randomized to receive either the pneumococcal (study) or the meningococcal (control) vaccine All infants will receive the glycoprotein conjugate pneumococcal vaccine.

Setting and Population:

This trial will be carried out in Dhaka, in collaboration with three private and public facilities providing maternity health care services. Pregnant women belonging to the middle-class come to these clinics for ante-natal care and delivery. Healthy pregnant women in the third trimester and their infants will be enrolled after signing informed consent forms. The study population will be pregnant women residing in Dhaka, Bangladesh. Our obstetrical co-investigators have helped us conduct our earlier studies with 180 women. They will again inform their registered patients of the purpose and of the trial, and refer them to our project staff assigned to that clinic. Project staff will provide a detailed explanation of the purpose, procedure, risk and benefits of the trial, answer any questions and then obtain signed informed consent of participation. Assignment to study groups will be instituted as soon as the mother gives concurrence to enroll into the study.

Selection criteria:

Inclusion criteria:

1. Women in third trimester of pregnancy with normal medical and obstetric history and physical examination and aged >15 and <40.
2. Women who plan to deliver in Dhaka and who plan to remain in Dhaka for the next 12 months.

Exclusion criteria:

1. Women with systemic disease as diabetes, hypertension, etc.
2. Women with known history of previous complicated pregnancy.
3. Women with previous history of preterm delivery and repeated abortions.
4. Women with previous history of birth with congenital anomalies.
5. Women with hypersensitivity reactions to vaccination.
6. Women with receipt of pneumococcal or meningococcal vaccine in last 3 years.

Study Procedure:

One hundred and eighty healthy pregnant women in their third trimester will be enrolled after informed consent. Each will be given a unique identification number, which will indicate the study group she is assigned to. The numbers assigned will be from a pre-prepared list of numbers. Randomization will be a one step block design of 4 to ensure that in each block the groups are equally represented. This will entail in the study groups will be randomised to the receiving the pneumococcal polysaccharide vaccine (study), and those in the control study groups meningococcal vaccine (25) . All infants will receive the conjugate pneumococcal vaccine at 6, 10 and 14 weeks of age (Appendix 1). Both these polysaccharide vaccines have been shown to be safe in our hands in published studies in Dhaka (13,25)

The design ensures that timing of blood specimens is parallel in the 4 groups, so direct comparison of each intervention can be made. For purposes of masking, every mother receives an injection of either pneumococcal or meningococcal vaccine. As such there will be total masking from the point of safety and side effect reporting in infant immunization. The major outcome variable (antibody levels) will also be determined by a completely blinded procedure.

The single dose vaccine vials will have a study label group code 1, 2, 3,4. The study coordinator will release vaccine vials to the study field staff who will take them to the obstetric practices. After determining the study group to which the mother is allocated, the field staff will note the study group allocation on the mothers' enrollment form, and write the mother's name and subject number on the vaccine vial label, and the vial number and study group in the mother's data form. After administration of vaccine, the empty study vaccine and tetanus vaccine vials will be returned to the coordinator, who will separately record the subject numbers and study group assignment against the vial numbers. Similar procedures will be followed for the study vaccines administered to the infants. The person who generates the allocation codes will not participate in subject assignment to study groups, vaccine administration, outcome assessment or data analyses, so that these processes remain blinded.

Baseline data collection

Data regarding demographic and socio-economic characteristics of the subjects will be recorded on the enrollment form. These data will include maternal age, height, years of education, gravidity, parity, date of the last menstrual period, number of adults in household, number of children in household, and father's years of education.

Immunization procedures

A. Immunization of mothers: The study vaccines will be administered intramuscularly in the deltoid region of the left arm of enrolled mothers, and all mothers will simultaneously receive tetanus toxoid vaccine in the right arm, as recommended by the Government of Bangladesh EPI program.

B. Immunization of infants: Infants will be assigned to the same study group as their mother, (see Appendix 1). All infants of enrolled mothers will receive the study vaccines at 6, 10 and 14 weeks of age, according to the schedule for their assigned group. At these routine immunization visits they

will simultaneously receive their standard immunizations, which consists of BCG, oral polio vaccine and DPT vaccine. The DPT vaccine will be administered in the left antero-lateral thigh.

C. Post Immunization Monitoring and Procedures: All mothers will be asked to wait in the health facility for 1-2 hours after immunization to monitor immediate side effects. Each enrolled mother will be given a questionnaire regarding possible local and systemic side effects at 24, 48, 72 hours after immunization. Subjects will have contact phone numbers of study team members and will report any reactions to the team. Every home will be visited at 72 hours to collect the reaction data. Data on the pregnancy and delivery will be obtained on all women. Data regarding illness during the pregnancy, the outcome of the delivery, estimated gestational age of the infant determined by the Ballard method (26) at delivery, the complications of delivery, birth weight, and neonatal illnesses will be recorded within 24 hours of delivery . Infants will be retained at the immunization clinic and observed for 1-2 hours after every immunization dose. Families will fill in reaction cards for 72 hours for fever and local and systemic reactions following each dose of vaccine. Study staff will visit the mothers and infants at home to collect information and nasopharyngeal specimens. Routine medical care and routine immunization will be provided for the infant at ICDDR,B clinics during the period of the study.

Vaccines: All vaccines in the study are US FDA licensed.

7- valent Conjugate pneumococcal vaccine (licensed in Feb 2000) is manufactured as a lyophilized preparation requiring reconstitution with saline aluminum phosphate diluent before use. Each 0.5 ml dose of reconstituted vaccine contains 2μg saccharide per serotype 4, 9V, 14, 18C, 19 F and 23F, and 4 μg of serotype 6B (20 μg total saccharide); approximately 20μg of CRM_{197} protein; and 0.5 mg of aluminum phosphate. This is a licensed product (Pnevenar®)of Wyeth-Ayerst.

23 valent pneumococcal polysaccharide vaccine contains 25 μg of each polysaccharide type dissolved in isotonic saline solution containing 0.25% phenol as a preservative. It is a mixture of highly purified capsular polysaccharides from 23 most prevalent or invasive pneumococcal types of streptococcus pneumoniae. This is a product (PNEUMOVAX 23) of Wyeth-Ayerst.

The **meningococcal vaccine** contains purified capsular polysaccharides of groups A,C,Y AND W-135 50 mcg of polysaccharide of each serotype. It is a product (Menomune ®) of Connought, used world-wide.

Specimens

See Appendix 1 for the schedule of collection of specific specimens, which will be collected at the follow up observation visits at birth, 6, 10, 14, 18-20 weeks.

Blood (5 ml) will be collected from mothers before and one month after immunization, and at the time of delivery. Infant cord specimens will be obtained at the time at delivery and infant (2ml) sera will be collected at 6, 14, and 18 weeks..

Breast milk will be collected at delivery and at 1, 6 and 18 weeks after delivery

Specimen processing Blood specimens will be centrifuged to separate serum, which will be aliquoted and frozen at -20 C at ICDDR, B until antibody assays are carried out. Breast milk specimens will also be centrifuged and the supernatant aliquoted and frozen at -20 C at ICDDR, B until antibody assays is carried out. All specimens will be labeled with the subject number and a specimen number, and their position in storage boxes will be recorded in the specimen database.

Microbiological processing of nasopharyngeal specimens for colonization study. Nasopharyngeal cultures for pneumococci will be obtained from the infants at birth and at each visit at 6, 10, 14, 18 weeks.

The nasopharyngeal swabs from the infants will be obtained in home or clinic using flexible Calgiswabs, immediately placed in Amies transport medium and shipped to the ICDDR, B microbiology laboratories and cultured on blood agar within 8 hours. We have standardized these procedures in Dhaka, and shown that pneumococci experimentally inoculated on Calgiswabs will survive for at least 2 days in local conditions in the Amies medium. The swabs will be inoculated in the laboratory on sheep blood agar with 0.5 g/ml gentamycin. Standard microbiological procedures will be used for identification and serotyping of *S. pneumoniae* isolates, using serotyping sera from Statens Seruminstitut, Copenhagen. Using these procedures, we have shown that up to 78% of Dhaka infants are colonized by pneumococci before 6 months of age.

Laboratory procedures assessment:

Sera and breast milk specimens will be separated, aliquoted, stored at -20°C at ICDDR,B and later shipped on dry ice for antibody assays. Serotype specific IgG and IgA (class and sub-class) antibodies to *S. pneumoniae* to 4 vaccine types, (1, 6, 19, 23) and 2 non- vaccine types, (2 and 42) will be assessed on serum and breast milk samples using the standard CDC/WHO standardized ELISA techniques) with minor modifications (27). Serum specimens from each mother-infant pair will be assayed together in a single plate. IgG1 and IgG2 antibodies to type 19F pneumococcal polysaccharide will be determined, if additional support for this purpose is obtained. The pneumococcal assays will be standardized against immune serum pool 89-SF (Dr. Carl Frasch, Center for Biologics Evaluation and Research, FDA) with the following antibody assignments: 13.4 μg/ml of IgG and 0.5 μg/ml of IgA anti-pneumococcus type 6B, 9.1 μg/ml of IgG and 0.6 μg/ml of IgA anti-type 19F. For IgG subclass assays serum 89-SF will be assigned 100 ELISA Units/ml since no μg/ml assignment has been established. Diphtheria and tetanus toxoid IgG antibody estimations will be by ELISA using methodology similar to those for the pneumococcal assays (27).

Outcome measures for primary hypothesis
The primary outcome measures are the infant serum IgG antibody titers to specific capsular pneumococcal polysaccharide, and to the protein carrier moiety of the pneumococcal conjugate vaccine. These data will be generated on coded specimens by the laboratory and reported as microgram per ml for each subject. The comparisons of interest are of the antibody concentrations at birth, which represents passively acquired maternal antibody; and the antibody concentrations after 3 doses of immunization at 18 weeks of age, which represent the active filial antibody response.

Sample size estimate

In order to estimate the sample size required to answer the primary research questions posed in the objectives of this proposal, we used standard sample size formulae and pneumococcal antibody geometric mean and standard deviation data from our studies of the 5 valent Praxis glycoprotein conjugate pneumococcal vaccine given to US infants at 2, 4 and 6 months of age (table 2 below).

Table 1 **ANTIBODY MEAN AND SD DATA**[a)]

serotype	N	IgG GMT	mean log	std. dev. of logs
19F	43	3.6 μg/ml	0.55882	0.605756
6B	45	2.3 μg/ml	0.36080	0.767809

[a)]values are for sera collected at 7 months of age, after 3 doses given at 2, 4 and 6 months of age to US infants

Table 2 utilizes the data in table 1 to show the number of subjects per group which are required to detect the specified differences between the antibody GMTs of groups 1 and 2. Since one goal of the project is to determine if a study group 2 has a lower antibody titer than group 1, a one-sided test of significance is appropriate, however, a 2-sided test allows full examination of the relationships.. A sample size of 63 in each group will allow us to detect a difference of 50% in the final geometric mean titers.

Table 2 **SAMPLE SIZE ESTIMATES**

group GMT (μg/ml)		Difference		Number each in group [a)]	
A	B	%	Fold	1 sided	2 sided
1.8	3.6	-50%	0.5	50	**63**
2.5	3.6	-30%	0.7	185	235
2.7	3.6	-25%	0.75	285	362
2.9	3.6	-20%	0.80	445	565

[a)] assuming alpha = 0.05, beta = 0.20, power = 80%.

We had 79% rate of completion in our previous study in Dhaka. If 30% of enrolled subjects do not complete the study, (63 ÷0.7 =) 90 subjects will need to be enrolled in each group, for a project total of 180 mother-infant pairs. (Appendix 3)

Using similar assumptions, evaluating 90 subjects per group will provide 90% power to detect a change in colonization rate between 2 groups from 80% to 50% or lower. 90 subjects will also allow 80% power to detect a correlation coefficient -0.35 or greater between 2 variables, and 95% power to detect a coefficient of -0.50 or greater.

Training and supervision of staff

A full time physician, study coordinator, data manager, health assistants and field workers will be employed for the field activities of this project. We will try to select experienced personnel, some of whom were involved in our previous study of maternal immunization in Dhaka. There will be extensive training sessions in the procedures for the project, focusing on the data to be gathered in the clinic and homes of the subjects. There will be extensive review and practice in filling in the data forms; selected health assistants will review the procedures of the Ballard gestational age scoring system in a hospital nursery with normal newborn infants. The scores determined independently by the health assistants will be compared with those determined by the PI (Dr. Shahid) and the study physician to assess the accuracy and consistency of the field workers examination.

Daily supervision of field staff will be done. To ensure consistency and quality of data collection and recording, and to determine a need for additional training of field staff, we will carry out routine checks and comparisons of data acquired by individual study personnel. For example, a 10% random sample of subjects will be visited and data forms filled out a second time. The accuracy of these data will be reviewed and retraining instituted.

Data management and quality control

The interview data will be recorded into the data forms by the field team at the time of interviewing the subjects. The completed data forms will be reviewed by the data manager who will determine that the forms are complete without blanks and obvious errors. On a weekly basis the forms will be double- entered into an EpiInfo database, once by one person, and again by another. At each entry range and logical checks will be performed. The two entered files then will be compared; inconsistencies will be resolved in consultation with the research coordinator and the PI. If necessary a specific query will go to the field worker who will contact the subject and resolve the inconsistency. Weekly frequency tables of variables will be reviewed by the PI to discover aberrant data. Quarterly summary tables will be shared with co-PI.

The laboratory data will be also doubled-entered into the data system. Summary tables of frequencies and ranges of antibody data will be reviewed to identify possibly anomalous values. Values, which appear anomalous or out of expected range will be referred to the serology laboratory for review. We will periodically resubmit a random selection of samples for independent assessment of assay variation. In addition, the laboratory has routine procedures for quality control which include periodic documentation of variance of the assay on standard sera.

Threats to validity

Lack of compliance with the intervention is unlikely to be a problem, because all interventions are an injection administered by study staff. Co-intervention in the form of receipt of study vaccines from other sources is also unlikely, since neither pneumococcal nor meningococcal vaccine are readily available in Dhaka. In order to determine the possibility of administration of routine BCG, DPT, OPV or other vaccine in study infants, we will question mothers at each visit if injections or immunization have been given to the child by non-study personnel. The provision of free care and routine immunizations to the infants makes co-intervention an unlikely possibility. Bias due to confounding factors and selection bias will be reduced by the randomization procedures.

Antibody assay validity and variability : Because antibody titers are the principal outcomes of this studies, we have instituted procedures to ensure validity and to reduce the variability of antibody assays. The ELISA for pneumococcal antibody has been standardized using the CDC/WHO standard assay procedures and FDA reference serum by Dr. Hamilton at Johns Hopkins University. Interassay variability is now assessed by running a local control serum (89-1C) on each plate to measure day to day variations. During a recent study of pneumococcal immunization of adults with and without HIV infection the co-efficients of variation for serotypes 6B, 14, 18C, 19F, and 23F were 30%, 12%, 11%, 12%, 16%, respectively . These values were typical for variation as reported by other centers, including the observation that type 6B has greater variability than the remaining serotypes. The laboratory will receive the serum coded and thus they will remain blinded to the code assignments.

Data analysis (see appendix 2)

Sera with undetectable antibody will be assigned half the lower limit of detection for the purposes of calculating means. Because the distributions of antibody titers and of the increases in titer (post-immunization antibody level divided by pre-immunization antibody level) are likely to be positively skewed, the values will be log-transformed to approximate a normal distribution for statistical analyses. The antilogarithms of the means of the log-transformed values will be reported as geometric mean titers (GMT) or as geometric mean increases. Continuous variables will be compared between groups by two-sided T-test or Mann-Whitney U test within groups by the paired T-test, and associations of continuous variables will be assessed by Pearson's correlation coefficient or the Spearman rank correlation test. The Chi Square or Fisher's exact test will be used to compare proportions between groups.

The gestational age of the infants will be estimated by two methods. The mothers will be asked the date of the first day of their last menstrual period, from which we will calculate the duration of the gestation period. In addition, each child will be examined within 48 hours of birth using the method of Ballard to assess the neuromuscular and physical maturity of the infant (**25**). The Ballard technique assigns a score for neuromuscular and physical maturation and allows an estimation of gestational age.

In order to determine the success of the randomization procedure, the following variables of the mothers and infants will be compared to determine if the means or proportions are significantly different between the groups: maternal age, height, education, and parity, household size, gestational age at immunization, gestational age at delivery, immunization-birth delivery, birth weight, percent of infants with birth weight less than 2.5 kg, and less than 37 weeks gestation. The geometric mean pneumococcal antibody titers before immunization and at the time of delivery will be compared between mothers in the study groups, by ANOVA. Similarly, the infants' antibody titers to pneumococcal polysaccharide, diphtheria toxoid and tetanus toxoid will be compared between the three study groups in cord, 6 week and 18 week specimens by ANOVA test. The groups will be compared with respect to key characteristics to assess randomization. Proportions with adverse events and proportions seroconverting will be compared using chi-square tests.

We will compare the final infant pneumococcal antibody GMT between study group 1 and 2 to assess the effect of passive antibody on the filial antibody response to three doses of glycoprotein conjugate pneumococcal vaccine.

The effect of specific maternal antibody on the filial immune response to pneumococcal conjugate vaccine will be assessed by calculating the correlation coefficient of infant antibody titers at 18 weeks (one month after the third dose of vaccine) with the specific antibody titer in the cord specimen (predictor variable). A negative correlation between the two would imply that higher antibody titers in the cord specimen inhibit the filial production of antibodies after active immunization. To determine if maternal antibody to the carrier protein (diphtheria toxoid) influences the filial antibody response to the glycoprotein conjugate vaccine or to diphtheria vaccine, the correlation of infant pneumococcal polysaccharide and diphtheria toxin antibody titers at 18 weeks with infant cord titers for both antigens will be examined. A negative correlation between cord diphtheria antitoxin antibody levels and final pneumococcal polysaccharide antibody would suggest inhibition related to maternal protein carrier antibodies. Similar analyses of correlation with pneumococcal polysaccharide antibodies will be done using tetanus toxoid antibodies as a control, since both mothers and infants receive tetanus vaccine. A multivariable regression technique will be used to assess the joint and separate effects of maternal antibody titers to pneumococcal polysaccharide, diphtheria and tetanus antigens, birth weight, immunization interval and other demographic variables on the final infant antibody titers to pneumococcal polysaccharide, tetanus and diphtheria toxoids. The natural decay of the antibody will be calculated based on rate of natural decay per week.

We will test the hypothesis that conjugate pneumococcal vaccine reduces acquisition and carriage of pneumococci in infants. The main outcome measure will be the acquisition by the study children of specific pneumococcal serotypes (VT) contained in the vaccine (for 7 valent Lederle conjugate: groups/types 4, 6B, 9V, 14, 18C, 19F, 23F). The overall proportion of infants who acquire VT, the median age of acquisition, and median duration of carriage of VT will be compared between the study and control groups with appropriate statistical tests. Kaplan-Meier survival curves will be used to compare the cumulative proportions of children who acquire vaccine serotypes at each time point during this study. Bi-variate analyses will be carried out to determine the association of selected risk factors (breast feeding, crowding, etc) and the acquisition and carriage of VT and non-VT by study children. A multi-variable analysis will be done to determine the independent effects of these risk factors. The association of carriage of drug resistant pneumococci with risk factors will similarly be analyzed with bivariate and multivariable analyses. The data on carriage of pneumococci will be analyzed to determine if established carriage of VT pneumococci is shortened or otherwise influenced by the receipt of pneumococcal vaccine. If carriage of VT is reduced in recipients of pneumococcal vaccine, it will be of interest to determine if carriage of non-VT but pathogenic serotypes (types 7, 15) is increased in vaccinees, if there are adequate numbers of isolates of these types.

The association of specific serum IgG and IgA antibodies to the commonly carried pneumococcal types 6, 19, 14 and 23 with carriage of these types will be examined by chi square and regression techniques, to determine if there is an statistical association, and if there is a specific level of serum antibody which predicts reduction of carriage. We will also compare mean serum antibody titers for the most common colonizing serotypes between colonized and non-colonized infants. In mothers

who breast feed their infants, mean breast milk antibody titers against common serotypes will be compared in colonized and non-colonized infants (Appendix1)

Ethical issues

The study protocol has been reviewed and approved by the Research and Ethical Review Committees of the ICDDR,B in Dhaka, Bangladesh, and will be reviewed by the Joint Committee for Clinical Investigation of Johns Hopkins Hospital in Baltimore, Maryland.

A **data safety and monitoring committee** composed of three Bangladeshi physicians not involved in the study will be constituted. The members of the committee will be provided with vaccine reaction and other data by the Principal Investigator periodically during the study, and will communicate by fax and phone with each other and with the PI and Co-PI. These procedures allowed timely review and feedback regarding data and possible safety issues in our previous study in Dhaka.

Tentative Time schedule

We estimate that it will require up to 24 months (2 years) to carry out this study. Our estimate for each activity in the table below is based on our experience with the previous study of maternal immunization. The dates given are subject to project approval at ICDDR,B and JHU, MOU between the two institutions and transfer of funds to ICDDR,B.

Activity		Duration	Sample Dates *
1.	Local project approvals, hiring of field staff	2 months	06/02 *– 07/02
2.	Recruitment of subjects	7 months	08/02 – 02/03
3.	Follow up of last infants	6 months	10/02 – 03/03
4.	Laboratory assays, data analysis	9 months	04/03 – 12/03
5.	Report and publication writing	4 months	01/04 – 04/04

Policy Implications:

The results of this enhancement of a planned study will provide a direct comparison of infant immunization strategy with or without maternal immunization. Although a number of strategies have been recommended, a direct comparison of safety and immunogenicity is needed to allow planning for further studies. The expected impact is additional safety and immunologic information on the strategies proposed by WHO to reduce early infant mortality from pneumococcal disease. The results of this study will allow planning for further assessment of that strategy which appears to be the most effective.

Collaborative Arrangement

This is a collaborative arrangement between the Public Health Sciences and the Laboratory Divisions at the International Centre for Diarrhoeal Diseases Research, Bangladesh (ICDDR,B) and the Departments of International Health and Allergy and Immunology the Johns Hopkins University, Baltimore, USA.

Budget Justification:

The PI is a full-time scientific staff of ICDDR,B and has the experience of conducting randomized controlled trials of ARI vaccines in mothers and children. This project will occupy up to 50% 0f her time depending on the needs at different points during the trial. Dr Mahbubur Rahman is a microbiologist in charge of the ARI laboratory at ICDDR,B. Dr. Rob Breiman is a senior epidemiologist and has extensive experience in vaccine studies and will advise on the conduct of the trial and data analysis. Dr. SE Arifeen is an epidemiologist and Head, Child Health Programme will be helping in the data analysis. He has extensive experience in field studies.

A full-time physician will monitor the side-effects of the conjugate pneumococcal vaccine, which will be used for the first time in very young infants. Enrolled infants will have blood drawn from their veins on 4 occasions.

To save cost on local travel, the study team will be using public transport for follow-up home visits and not the ICDDR,B pool vehicles. International travel provisions are proposed for setting up the collaborative activities, data analysis and conference presentation.

The costs of microbiology and the immunology laboratories have been calculated. There will be about 1260 nasopharyngeal cultures done together with sensitivity and serotyping of pneumococcal strains. There will be 1260 blood samples from the study subjects. Pneumococcal antibodies i. e. measurements of total IgG, and specific subclass IgG_1 and IgG_2 will be done in individual samples. Functional antibodies will be done on selected samples. Serotype specific antibody assays will be conducted on 4 four vaccine types and 2 non-vaccine types.

Dissemination and Use of Findings:

Results of the study will be written up for publication in a peer reviewed journal and presented at International and Regional scientific conferences. We shall share the results of this investigation immediately with our collaborating investigators.

References:

1. JP Grant. The State of the World's Children 1995. UNICEF Report. Oxford University Press.
2. Kirkwood BR, Gove S, Rogers S *et al.* Potential interventions for the prevention of childhood pneumonia in developing countries: a systematic review. Bull WHO 1995;73:793-89.

3. WHO/VRD/GEN/96.02. Review of immunization approaches to prevent diseases occurring in early childhood.
4. Shann F. Etiology of severe pneumonia in children in developing countries. Ped Infect Dis 1984; 5: 247-52.
5. Gray BM, Converse III GM and Dillon Jr. HC. Epidemiologic studies of *Streptococcus pneumoniae* in infants: Acquisition, carriage and infection during the first 24 months of life. J infect Dis 1980; 142: 923-33.
6. Selwyn BJ. The epidemiology of acute respiratory tract infection in young children: comparison of findings from several developing countries. Rev Infect Dis 1990;12: S870-888.
7. Rahman M, Huq F, Sack DA *et al.* Acute lower respiratory tract infections in hospitalized patients with diarrhoea in Dhaka, Bangladesh. Rev Infect Dis 1990; 12: S899-906.
8. The WHO young Infant study Group. Bacterial etiology of serious infections in young infants in developing countries: results of a multicentre study. Pediatr Infect Dis J 1999; 18: S17-22.
9. Zaman K, Baqui AH, Yunus M *et al.* Acute respiratory infections in Children -A community based longitudinal study in Rural Bangladesh. J Trop Pediatrics 1997; 43: 133-37.
10. Mulholland K. Levine O, Nohynek H, Greenwood BM. Evaluation of vaccines for the prevention of pneumonia in children in developing countries. Epidemio Rev. 1999; 21: 43-55.
11. Saha SK. Rikitomi N. Ruhulamin M et al.. Antimicrobial resistance and serotype distribution of *Streptococcus pneumoniae* strains causing childhood infections in Bangladesh. J Clin Microb1999; 37: 798-800.
12. Fedson DS. The clinical effectiveness of pneumococcal vaccination: a brief review. Vaccine 1999;17:S85-90.
13. Shahid NS, Steinhoff MC, Hoque SS *et al.* Serum breast milk and infant antibody after maternal immunization with pneumococcal vaccine. Lancet 1995; 346:1252-57
14. Panpitpat C, Thisyakorn U, Chotpitayasunondh T et al. Elevated levels of maternal anti-tetanus toxin antibodies do not suppress the immune response to a *Haemophilus influenzae* type b polyribosyl-phosphate-tetanus toxoid conjugate vaccine. Bull WHO, 2000;78: 364-371.
15. Siber GR. Pneumococcal Disease: Prospects for a new generation of vaccines. Science 1994; 265: 1385-87.
16. Shann F. Pneumococcal vaccine: time for another controlled trial. Lancet 1998; 351;1600-1.
17. Fischer GW, Ottolini MG, Mond JJ. Prospects of vaccines during pregnancy and in the newborn period. Clin Perinatal 1997;24:231-49.
18. Steinhoff MC, Edwards K, Keyserling H, *et al.* A randomized comparison of three bivalent Streptococcus pneumoniae glycoprotein conjugate vaccines in young children: effect of polysaccharide size and linkage characteristics. Ped Infect Dis J 1994;13:368-372.
19. Leach A, Ceesay SJ, Banya WA, Greenwood BM. Pilot trial of a pentavalent pneumococcal polysaccharide / protein conjugate vaccine in Gambian infants. Ped Infect Dis J 1996;15:333-6.
20. Daum RS, Hogerman D, Rennels MB, Bewley K et al. Infant immunization with pneumococcal CRM 197 vaccines: Effect of saccharide size on immunogenicity and interactions with simultaneously administered vaccines. JID;1997:176:445-455.
21. Obaro SK, Huo-Z, Banya-WA et al. A glycoprotein pneumococcal conjugate vaccine primes for antibody responses to a pneumococcal polysaccharide vaccine in Gambian children. Pediatr Infect Dis J. 1997;16:1135-40.
22. Dagan R. Melamed R, Zamir O, Leroy O. Safety and immunogenicity of tetravalent pneumococcal vaccines containing 6B, 14, 19F and 23F polysaccharides conjugated to either tetanus toxoid or diphtheria toxoid in young infants and their booster ability by native polysaccharide antigens. Pediatr Infect Dis J. 1997;16:1053-9.

23. King JC Jr, Vink-PE, Farley JJ, et al. Safety and immunogenicity of three doses of a five-valent pneumococcal conjugate vaccine in children younger than two years with and without human immunodeficiency virus infection. Pediatrics. 1997; 99:575-80.
24. Anderson EL, Kennedy DJ, Geldmacher KM, *et al.* Immunogenicity of heptavalent pneumococcal conjugate vaccine in infants. J.Pediatr 1996;128 (5.1):649-53.
25. Shahid NS, Steinhoff MC, Roy E, Thompson C and Begum T. Placental and breast milk transfer of antibodies after maternal immunization with polysaccharide meningococcal vaccine: a randomized controlled evaluation. (in press).
26. Ballard JL, Novak KK, Marshall Driver BA. A simplified score for assessment of foetal maturation of newly born infants. Journal of Paediatrics. 1979;95:769-774
27. Human IgG, IgA and IgM anti-Pneumococcal capsular polysaccharide antibody standard ELISA protocol. WHO ELISA Workshop, CDC consensus assay. Geneva: World Health Organization 1996.

Appendix 1 **SCHEDULE OF PROCEDURES**

	MOTHER (M)			INFANT (1)				
**Groups**	**32 wks**	**1 mth**	**Delivery**	**Birth (0 wks)**	**6 wks**	**10 wks**	**14 wks**	**18 wks**
**1 (routine I)**	Mn,M	M	M	B	CjSp,B	CjSp,B	CjSp,B	B
**2 (M+I)**	Ps,M	M	M	B	CjSp,B	CjSp,B	CjSp,B	B

NP cultures at each contact

Ps: 23-valent polysaccharide pneumococcal vaccine,
Mn: polysaccharide meningococcal vaccine (control)
CjSp: pneumococcal conjugate vaccine

M - Maternal blood specimens
B - Infant blood specimens,

NP- nasopharyngeal cultures for *S. pneumoniae* isolation and serotyping

Appendix 2 **SUMMARY OF ANALYSES**

Objectives	Variables	Analysis
**Primary: 1a** Immunogenicity	antibody titer: GMT, % > 0.15, 1µg/ml	t-test, chi square regression
**Primary: 1b** Safety	% reaction rates	chi square
**Secondary: 2** Maternal-filial	cord, final antibody: GMT, % >0.15, 1 µg/ml	correlation coefficient
**Secondary: 3** EPI schedule	cord , final antibody; GMT; GMT, % >0.15, 1 µg/ml	means, proportions
**Secondary: 4** NP colonization	cord , final antibody; GMT; % colonization rates	chi square, regression

Appendix 3 Two-Sample T-Test Power Analysis

Numeric Results for Two-Sample T-Test
Null Hypothesis: Mean1=Mean2. Alternative Hypothesis: Mean1>Mean2
The standard deviations were assumed to be known and equal.

Power	N1	N2	Allocation Ratio	Alpha	Beta	Mean1	Mean2	S1	S2
0.80286	63	63	1.00	0.02500	0.19714	0.559	0.258	0.601	0.601
0.80514	50	50	1.00	0.05000	0.19486	0.559	0.258	0.601	0.601

Two-Sample T-Test Power Analysis
Numeric Results for Two-Sample T-Test
Null Hypothesis: Mean1=Mean2. Alternative Hypothesis: Mean1>Mean2
The standard deviations were assumed to be known and equal.

Power	N1	N2	Allocation Ratio	Alpha	Beta	Mean1	Mean2	S1	S2
0.80000	40	40	1.00	0.02500	0.20000	0.559	0.182	0.601	0.601
0.80000	50	50	1.00	0.02500	0.20000	0.559	0.222	0.601	0.601
0.80000	60	60	1.00	0.02500	0.20000	0.559	0.251	0.601	0.601
0.80000	70	70	1.00	0.02500	0.20000	0.559	0.274	0.601	0.601
0.80000	80	80	1.00	0.02500	0.20000	0.559	0.293	0.601	0.601
0.80000	90	90	1.00	0.02500	0.20000	0.559	0.308	0.601	0.601
0.80000	100	100	1.00	0.02500	0.20000	0.559	0.321	0.601	0.601
0.80000	40	40	1.00	0.05000	0.20000	0.559	0.225	0.601	0.601
0.80000	50	50	1.00	0.05000	0.20000	0.559	0.260	0.601	0.601
0.80000	60	60	1.00	0.05000	0.20000	0.559	0.286	0.601	0.601
0.80000	70	70	1.00	0.05000	0.20000	0.559	0.306	0.601	0.601
0.80000	80	80	1.00	0.05000	0.20000	0.559	0.323	0.601	0.601
0.80000	90	90	1.00	0.05000	0.20000	0.559	0.336	0.601	0.601
0.80000	100	100	1.00	0.05000	0.20000	0.559	0.347	0.601	0.601

M2 vs N1 by Alpha with M1=0.559 S1=0.601 S2=0.601
Power=0.80 N2=N1 1-Sided T Tes

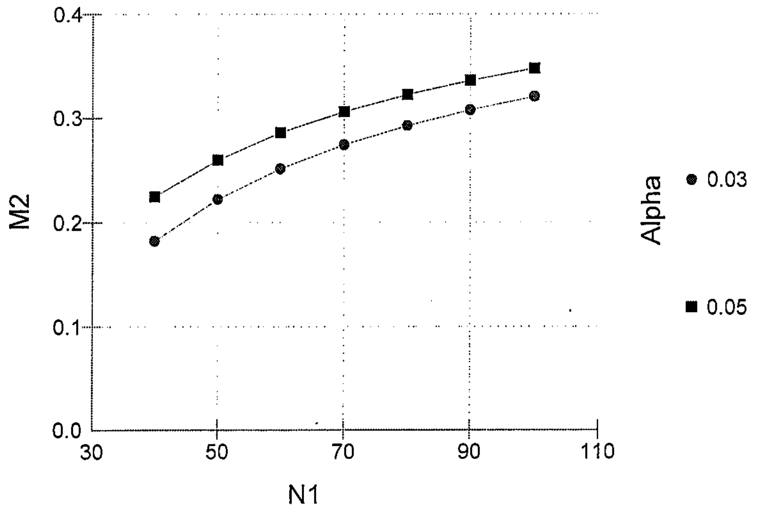

Supplement: Protocol S1 — Trial protocol. (PDF) [file pone.0070867.s002.pdf]
